# α-Tubulin acetylation at lysine 40 regulates dendritic arborization and larval locomotion by promoting microtubule stability in *Drosophila*

**DOI:** 10.1371/journal.pone.0280573

**Published:** 2023-02-24

**Authors:** Xiaoxiao Niu, Chuan-Xi Mao, Shan Wang, Xiongxiong Wang, Youyu Zhang, Juncheng Hu, Ran Bi, Zhihua Liu, Jin Shan

**Affiliations:** State Key Laboratory of Biocatalysis and Enzyme Engineering, Hubei Province Key Laboratory of Biotechnology of Chinese Traditional Medicine, National & Local Joint Engineering Research Center of High-throughput Drug Screening Technology, School of life science, Hubei University, Wuhan, China; University of Illinois at Chicago, UNITED STATES

## Abstract

Posttranslational modification of tubulin increases the dynamic complexity and functional diversity of microtubules. Acetylation of α-tubulin at Lys-40 is a highly conserved posttranslational modification that has been shown to improve the flexibility and resilience of microtubules. Here we studied the *in vivo* functions of α-tubulin acetylation by knocking-out Atat, the *Drosophila* α-tubulin acetyltransferase, and by mutating Lys-40 to Arg in α1-tubulin. We found a reduction in the dendritic arborization of larval class I dendritic arborization (da) neurons in both mutants. The dendritic developmental defects in *atat* mutants could be reversed by enhancing the stability of microtubules either through knocking down the microtubule severing protein Katanin 60 or through overexpressing tubulin-specific chaperone E, suggesting that α-tubulin deacetylation impairsed dendritic morphology by decreasing the stability of microtubules. Using time-lapse recordings, we found that *atat* and *α1*-*tubulin*^*K40R*^ mutations dramatically increased the number of dendritic protrusions that were likely to be immature dendritic precursors. Finally, we showed that both Atat and α-tubulin acetylation were required in class I da neurons to control larval locomotion. These findings add novel insight into the current knowledge of the role of α-tubulin acetylation in regulating neuronal development and functions.

## Introduction

Post-translational modifications (PTMs) occur on microtubules, regulating their structure, dynamics, and a variety of functions [1, 2]. Acetylation of α-tubulin is an important PTM. The main acetylation site in α-tubulin is lysine 40 (K40) in the microtubule lumen [3, 4]. Acetylation of α-tubulin was initially characterized as a marker of microtubules that are resistant to depolymerizing drugs and is typically correlated with stable, long-lived microtubules in cells [5–7]. The level of microtubule acetylation significantly affects the function and morphogenesis of neuronal terminals, axonal transport, neuronal development, and neuron migration [1, 8–10]. Treatment with tubacin, a specific inhibitor of the tubulin deacetylase activity of histone deacetylase 6 (HDAC6), increases the level of microtubule acetylation and promotes axonal vesicular transport [8]. In contrast, a decrease in microtubule acetylation leads to abnormal development and migration of neurons projecting into the cerebral cortex of rats [10]. Moreover, microtubule acetylation is associated with many neurodegenerative diseases, such as Charcot-Marie-Tooth disease, Alzheimer’s disease, and Huntington’s disease [11–13]. Inhibition of HDAC6 restores tau-induced microtubule defects and abnormal development of the neuromuscular junction (NMJ) [14]. Thus, the dynamic regulation of microtubule acetylation is important for neuronal development, its dysregulation is a risk factor for neurodegenerative diseases.

The α-tubulin *N*-acetyltransferase 1 (αTAT1)/MEC-17 is a protein related to Gcn5 histone acetyltransferases and acts as a K40-specific acetyltransferase for α-tubulin. *In vitro*, αTAT1 exclusively acetylates α-tubulin K40 ☯15]. αTAT1 is conserved from *Tetrahymena* to mammalian species and is the major α-tubulin acetyltransferase to promote α-tubulin acetylation [15, 16]. Numerous reports have shown that αTAT1 is actively expressed and plays an important role in the neuronal system. Disrupting the *αTAT1* gene results in the formation of short polymorphic microtubules in touch receptor neurons and progressive axonal degeneration, axonal transport disorders, and alterations in presynaptic protein distribution in *Caenorhabditis elegans* [17, 18]. In rats, *αTAT1* deficiency causes migratory defects in the cortical projection neurons and interneurons and perturbs the transition of projection neurons from the multipolar stage to the unipolar/bipolar stage in the intermediate zone of the cortex as well [10]. The αTAT1-mediated α-tubulin acetylation restrains axon branching and growth by dampening microtubule plus-end dynamics in the mouse central nervous system and *atat*-deficient mice demonstrate deformed dentate gyrus, suggesting that αTAT1 may be important for advanced cognitive functions such as learning and memory [19, 20]. The *atat* mutation also results in defects in sensory neuron development. Lacking αTAT1 activity in *C*. *elegans* results in touch insensitivity [15, 16, 21], and *atat*-deficient mice are also insensitive to mechanical touch and pain [22]. In *Drosophila*, Atat-regulated α-tubulin acetylation is required for the activation of mechanosensory channels upon mechanical stimulation in peripheral sensory neurons [23].

Here, we extend our knowledge of the functions of tubulin acetylation in regulating the dendritic development of sensory neurons. Using mutants with α-tubulin lysine 40 mutated and newly generated *atat* mutants, we show that depleting tubulin acetylation results in reduced dendritic arborizations of a specific type of sensory neuron. Importantly, defects in dendritic development can be fully rescued by enhancing microtubule stability. Thus, our results demonstrate that α-tubulin K40 acetylation promotes dendritic arborization by maintaining the stability of the microtubule cytoskeleton.

## Material and methods

### *Drosophila stocks* and husbandry

Flies were cultured on standard cornmeal medium at 25°C, *w*^*1118*^ was used as the control unless otherwise specified. The following fly lines were purchased from Bloomington *Drosophila* Stock Center: the muscle-specific driver *C57-Gal4* [24], pan-neuronal driver *elav-Gal4* (Bloomington Stock number, 458), class I sensory neuron-specific driver *221-Gal4* [25], and *UAS-mCD8-GFP* (Kyoto Stock number, 108068). The *Katanin 60* RNAi line (v38369) was obtained from Vienna *Drosophila* Resource Center. *UAS-tubulin-specific chaperone E* (*tbce*), *α1-tubulin*^*K40Q*^, and *α1-tubulin*^*K40R*^ were as described previously [26, 27].

### Generation of *UAS-atat* transgenic flies

The full-length cDNA of *atat* (NM_140052) was cloned into *pUASTattB* [28]. Subsequently, *pUASTattB*-*atat* was integrated into the second chromosome of *ZH-attP-51D* flies (Bloomington Stock number, 24483) at 51D using site-directed integration [29].

### Generation of *atat* mutants

The *atat*^*15*^ and *atat*^*16*^ alleles were engineered using CRISPR/Cas9-mediated targeted mutagenesis [30]. Two sgRNAs were designed: sgRNA1 (caatcggggtgcagcataatg) recognized the DNA sequence near the start codon of *atat* and sgRNA2 (gttcgcgcagccaatcatcaagg) targets the 33rd to 55th bp of the first exon. These two sgRNAs were cloned into the *pCFD3-U6b* separately and were co-injected into *nos-Cas9* (Bloomington Stock number, 54591) embryos.

### Western blot analysis

Western blot analysis was performed as described previously [26, 27]. Third instar larvae were dissected in PBS, followed by homogenization in a lysis buffer (50 mM Tris-HCl, pH 7.4, 150 mM NaCl, 1% NP-40, and 0.1% SDS). Blots were first probed with primary antibodies, anti-α-tubulin (1:50,000; mAb B-5-1-2; Sigma-Aldrich), anti-acetylated-tubulin (1:10,000; mAb 6-11B-1; Sigma-Aldrich), and anti-actin (1:50,000; mAb 1501; Millipore), followed by incubation with horseradish peroxidase (HRP)-coupled secondary antibodies (1:50,000; Sigma-Aldrich). Protein bands were visualized using a chemiluminescence method (ECL Kit, Amersham). The intensities of positive bands were measured in ImageJ.

### Immunofluorescence and confocal microscopy

Dissection and antibody staining were performed as described previously [26, 31]. Primary antibodies included: rabbit anti-vasa (1:8,000; provided by Dr. Z. Wang) [32], fluorescence-conjugated anti-HRP (1:200; Jackson ImmunoResearch), mouse anti-α-tubulin (1:1,000; mAb B-5-1-2; Sigma-Aldrich), rabbit anti-α-tubulin (1:200; ab18251; Abcam), mouse anti-acetylated tubulin (1:800; mAb 6-11B-1; Sigma-Aldrich), anti-GFP (1:500; 50430-2-AP; Proteintech), and anti-Futsch [1:50; 22C10; the Developmental Studies Hybridoma Bank at the University of Iowa]. Nuclei were visualized by staining with TO-PRO(R) 3 iodide (1:1000; Invitrogen) for 2 hours at room temperature. Images were collected with a Zeiss 710 confocal microscope. All images analyzed were projections from complete Z-stacks.

### Quantification of tubulin and acetylated tubulin in different tissues

**Fig 1:**
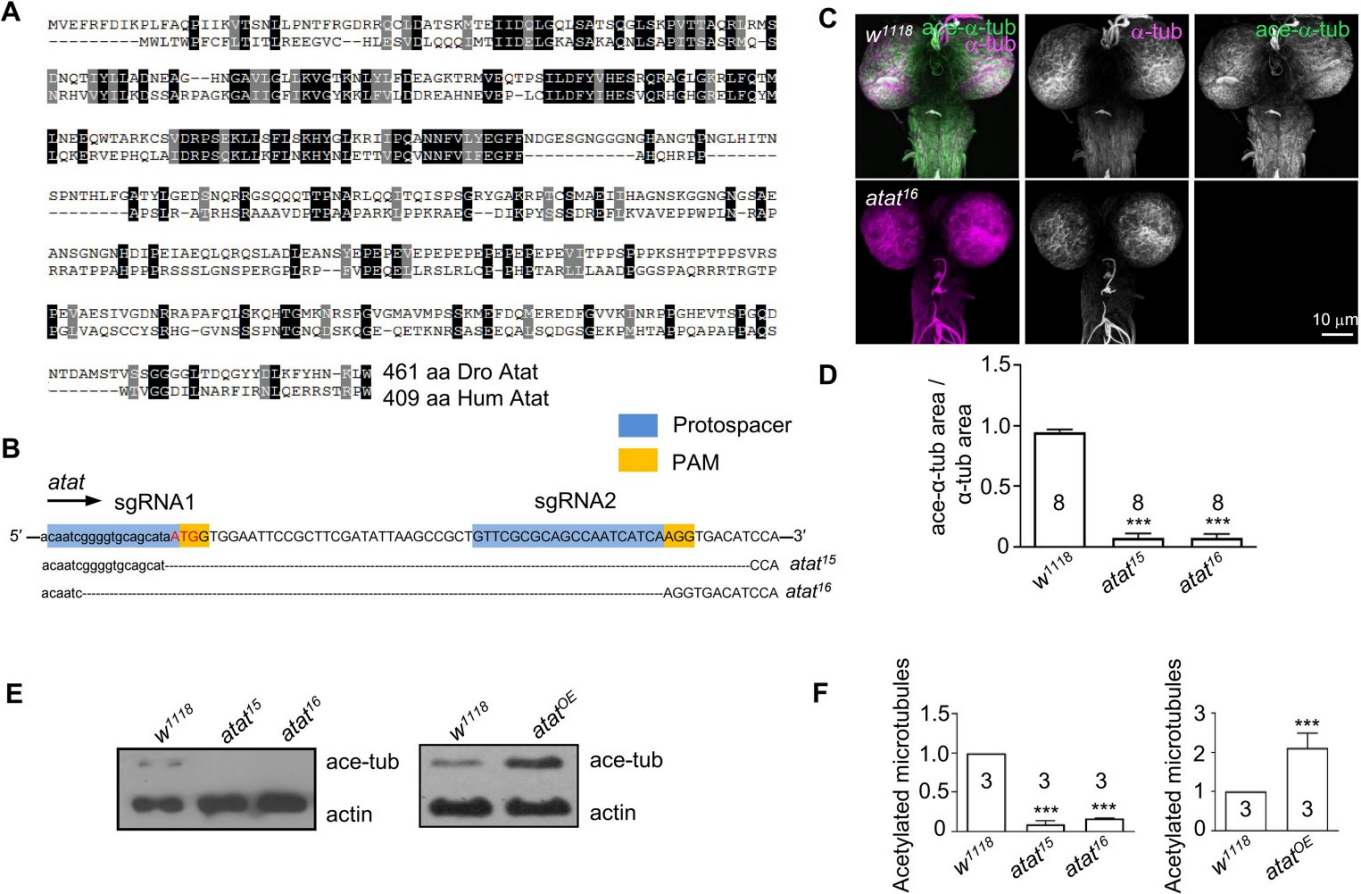
Atat is the major α-tubulin acetyltransferase in the *Drosophila* nervous system. (A) Sequence alignment of *Drosophila* Atat (Dro Atat) and human αTAT1 (Hum Atat). Black and gray shading indicate identical (25%) and similar amino acids (40%), respectively. (B) The Cas9/sgRNA-targeting sites were designed to disrupt the coding regions of *atat* and the sequencing results of the deleted regions in two *atat* mutants. The *atat* UTR sequence is presented in lowercase and the start codon is presented in red uppercase. The *atat* coding sequence is presented in uppercase. (C) The larval nervous systems of control (*w*^*1118*^) and *atat*^*16*^ mutants are double-labeled with anti-α-tubulin (green) and anti-acetylated tubulin (magenta). Scale bar: 10 μm. (D) Normalized acetylated microtubule density in wildtype control and two *atat* mutants. Mean ± SEM. One-way ANOVA with Tukey’s multiple comparisons test. ***p < 0.001. (E) Western analysis of acetylated α-tubulin in the larval nervous system in control (*w*^*1118*^), *atat* mutants, and larvae with Atat overexpressed using *elav-Gal4* (*atat*^*OE*^). Actin was used as a loading control. (F) Normalized intensities of acetylated microtubules. Mean ± SEM. One-way ANOVA with Tukey’s multiple comparisons test. ***p < 0.001.

**Fig 2:**
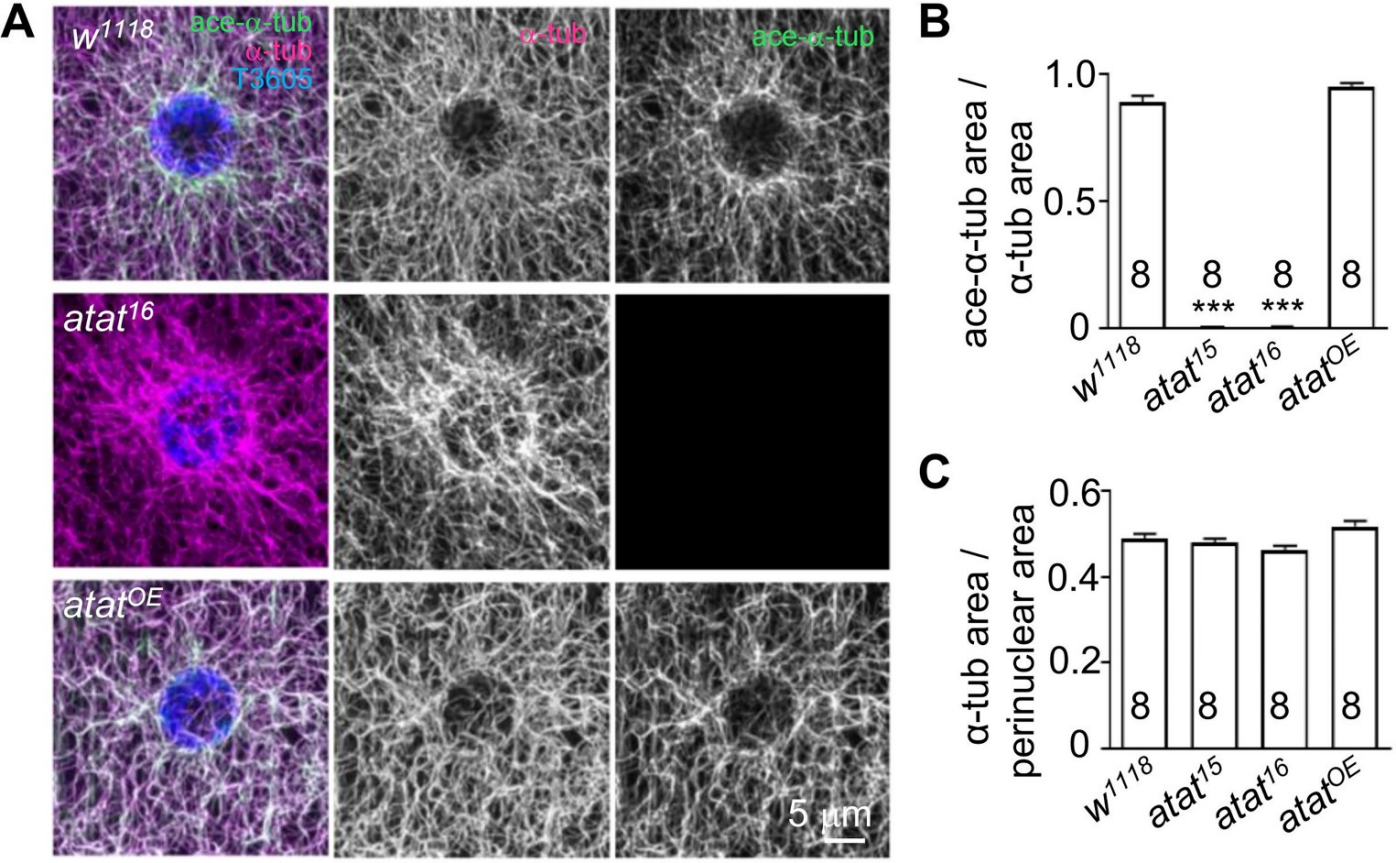
Depleting α-tubulin acetylation in muscle cells has a minor effect on the microtubule network. (A) The larval muscle cells of the control (*w*^*1118*^), *atat*^*16*^ mutant, and larva with Atat overexpressed using *C57-Gal4* (*atat*^*OE*^) were triple-stained with anti-α-tubulin (green), anti-acetylated α-tubulin (magenta), and TO-PRO(R) 3 iodide (T3605, blue). Scale bar: 5 μm. (B and C) Quantification of the densities of acetylated microtubules (B) and total microtubules. Mean ± SEM. One-way ANOVA with Tukey’s multiple comparisons test. ***p < 0.001.

**Fig 3:**
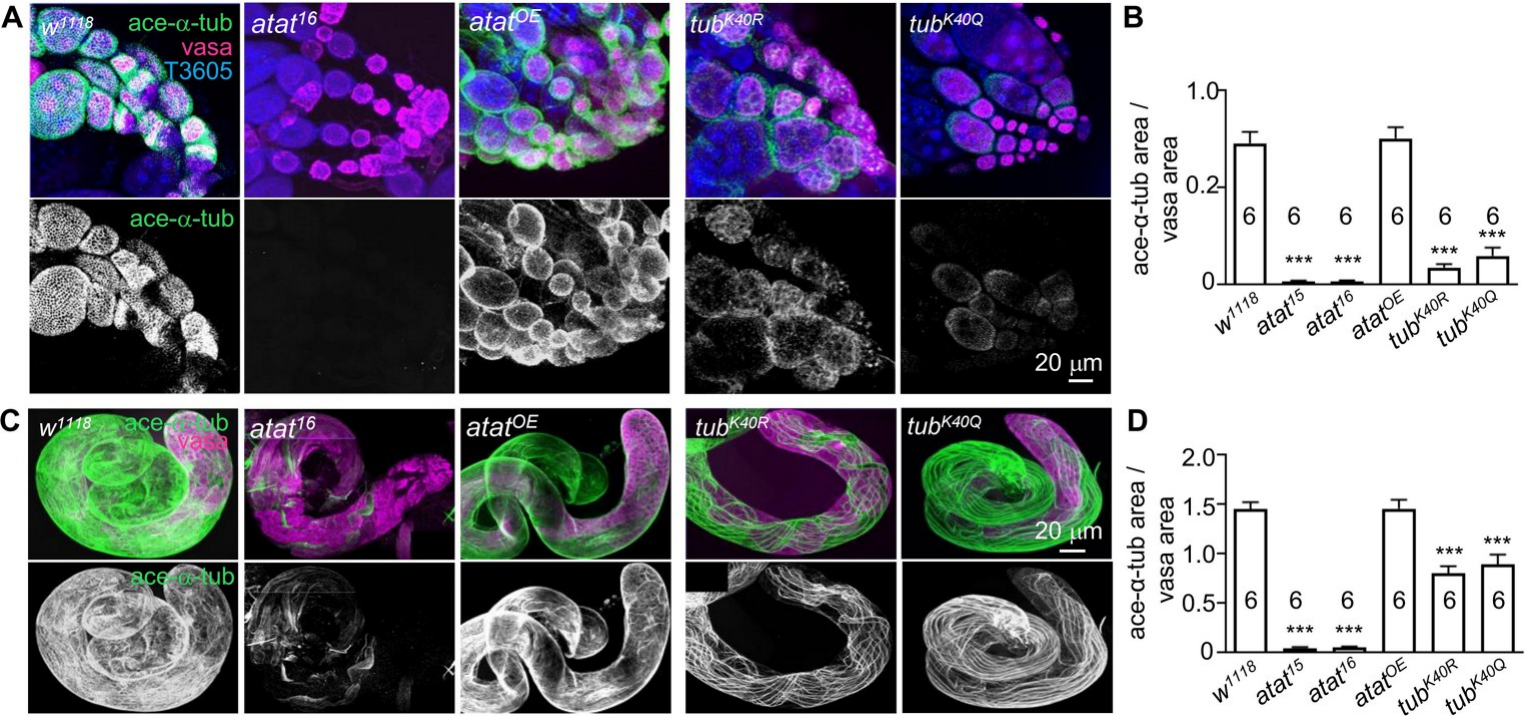
Atat is not the sole α-tubulin acetyltransferase in the adult testes. (A) Triple-staining of adult ovaries of control (*w*^*1118*^), *atat*^*16*^, and Atat overexpression driven by *vasa-Gal4* (*atat*^*OE*^), *α1-tubulin*^*K40R*^ (*tub*^*K40R*^), and *α1-tubulin*^*K40Q*^ (*tub*^*K40Q*^) with anti-acetylated α-tubulin (green), anti-vasa (magenta, labels germ cells) and DNA dye TO-PRO(R) 3 iodide (T3605, blue). Scale bar: 20 μm. (B) Quantification of acetylated microtubule densities. Mean ± SEM. One-way ANOVA with Tukey’s multiple comparisons test. ***p < 0.001. (C) Adult testes stained with anti-acetylated tubulin (green) and anti-vasa (magenta) to show microtubule network and germ line cells. Scale bar: 20 μm. (D) Quantification of acetylated microtubule densities. Mean ± SEM. One-way ANOVA with Tukey’s multiple comparisons test. ***p < 0.001.

**Fig 4:**
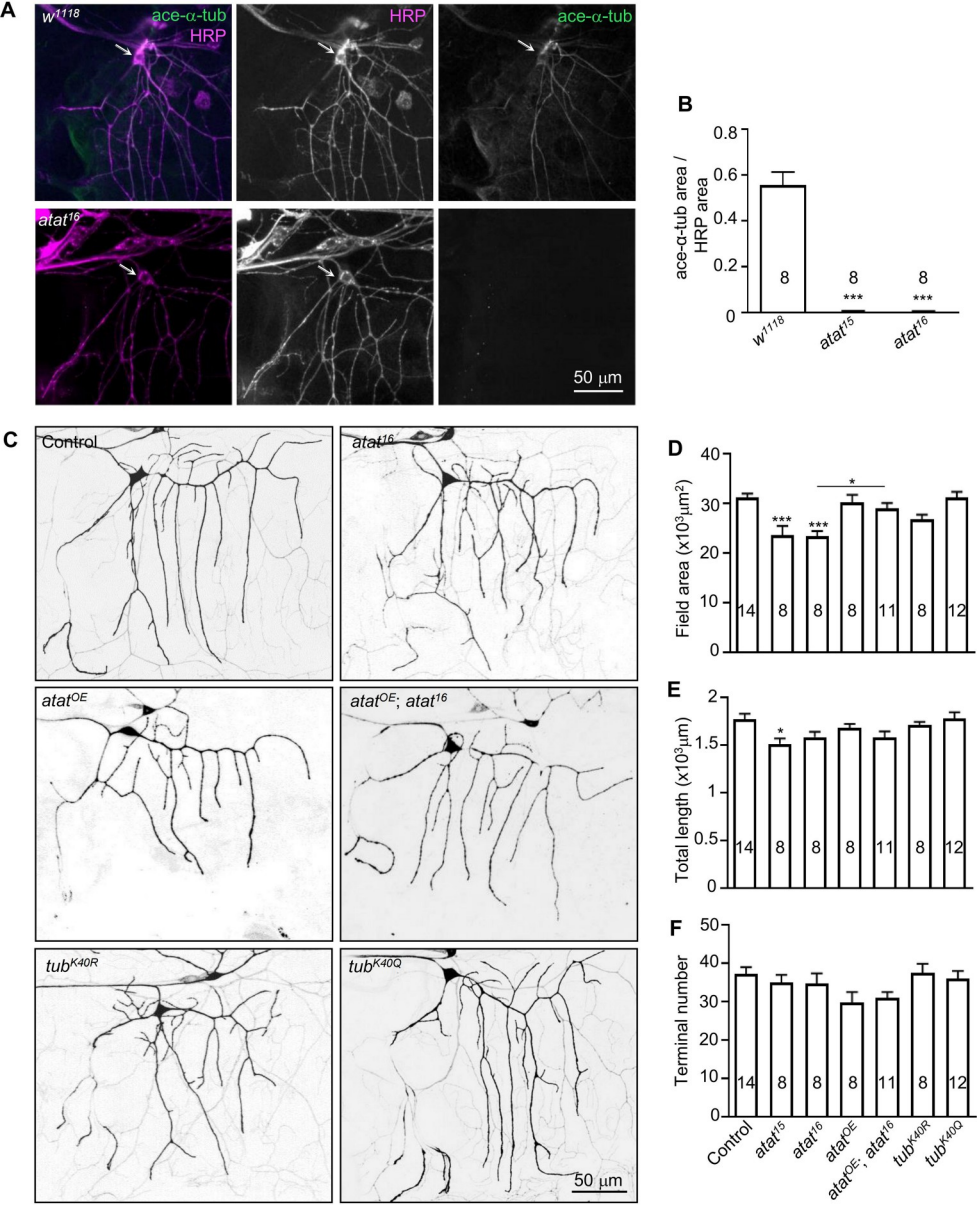
Acetylated α-tubulin is required for dendritic arborization of class I da sensory neurons. (A) Class I da neurons in both control (*w*^*1118*^) and *atat*^*16*^ mutant were stained with anti-acetylated tubulin (green) and anti-HRP (magenta, labels neuronal membranes). (B) Quantification of acetylated microtubule levels in wild type and two *atat* mutants. Mean ± SEM. One-way ANOVA with Tukey’s multiple comparisons test. ***p < 0.001. (C) Dendritic elaboration of class I da neurons labeled with mCD8-GFP under the control of *221-Gal4*. The genotypes are control (*221-Gal4*>mCD8-GFP), *atat*^*16*^, Atat overexpression driven by *221-Gal4* (*atat*^*OE*^), Atat overexpression using *221-Gal4* in *atat*^*16*^ mutant (*atat*^*OE*^; *atat*^*16*^), *α1-tubulin*^*K40R*^ (*tub*^*K40R*^), and *α1-tubulin*^*K40Q*^ (*tub*^*K40Q*^). Scale bar: 50 μm. (D–F) Quantification of dendritic field areas (D), the total dendritic lengths (E), and the numbers of dendritic termini (F). Mean ± SEM. One-way ANOVA with Tukey’s multiple comparisons test. ***p < 0.001, *p < 0.01.

Quantification of tubulin and acetylated tubulin was performed largely according to our published protocol [31]. Images used for quantification were maximum intensity projections of Z-stacks for the sections that cover the whole larval nervous system (Fig 1), the nuclei of the muscle cells (Fig 2), the testis (Fig 3), the ovaries (Fig 3), and class I da sensory neurons (Fig 4). The area of the signals was estimated by setting a threshold using ImageJ and the same thresholds were used for all images from the same experiment. The results were expressed as the ratio of the acetylated tubulin-positive area divided by the area of control signals.

### Quantification of dendritic morphology of class I da sensory neurons

Maximum intensity projections of confocal images of class I da sensory neurons in abdominal segment 3 were used for quantification. A dendritic field is defined as the polygon delineated by connecting the distal-most dendritic tips of a da neuron [33]. The dendritic field areas, the lengths of dendritic branches, and the number of dendritic termini were measured manually in ImageJ.

### Live imaging analysis of dendritic branching events

Live imaging of dendritic branching events was performed largely according to previously published procedures [34]. Second instar larvae were anesthetized under 90% glycerol for 10 min in an enclosed Petri dish containing 3 ml of ether applied to a cotton ball. Anesthetized larvae were then transferred to a slide containing halocarbon oil 27 (Sigma-Aldrich) and covered with a coverslip that was gently depressed dorsally. Class I ddaE neurons from abdominal segment 3 were imaged on a Zeiss 710 confocal microscope using a 63× oil immersion objective. Z-stack images of terminal branches near the dorsal midline were collected approximately every 10 s for 10 min periods.

### Behavior assays

#### Larval locomotion

After a 60-second acclimation period on a 6.8-cm Petri dish filled with 1.5% agar, individual crawling third instar larvae were recorded for 60 seconds at 25 frames per second. Only continuous forward locomotion was used for quantitative analysis. Stride frequency was calculated by dividing the number of peristaltic contractions during a 12-second period (counted manually) by the number of seconds. Stride size was defined as the average distance traveled during one contraction [35]. To generate the plots in Fig 7B, the changes in body lengths during crawling were calculated using the “fit ellipse” function in ImageJ.

#### Gentle touch

The larval behavioral response to gentle touch was performed as previously described [36]. Each third instar larva was touched with an eyelash at one side of the thoracic segments during a bout of linear locomotion, and the behavioral responses were scored as follows: 0, no response; 1, pause; 2, recoil or turn; 3, single reverse contractile wave; and 4, retreat with multiple contractile waves. The response of each larva to gentle touch was tested four times and the touch scores were summed to obtain a score between 0 and 16.

### Statistical analyses

All statistical comparisons were performed using GraphPad InStat 5 software. P-values were calculated by one-way ANOVA. Comparisons were made between a specific genotype and the wildtype control (indicated by asterisks located above a column) or between two specific genotypes (indicated by asterisks located above a bracket).

## Results

### Atat is the major α-tubulin K40 acetyltransferase in the *Drosophila* nervous system

Recent studies have shown that Atat is a *Drosophila* homology of the human αTAT (**Fig 1A**), and required in the larval peripheral nervous system for mechanosensation [23] and in the larval motor neurons for the growth of axonal terminal boutons [37]. We generated two new *atat* knockout alleles, *atat*^*15*^ and *atat*^*16*^, by CRISPR technology (**Fig 1B**). The *atat*^*15*^ allele contains a deletion from the first base before the start codon to the 61st base of the first exon of *atat*. Whereas, *atat*^*16*^ contains a deletion from the 13th base before the start codon to the 53rd base of the first exon of *atat*. We detected no acetylated α-tubulin signal in the larval ventral nerve cord of *atat* mutants by either immunostaining (**Fig 1C and 1D**) or western blot analysis (**Fig 1E and 1F**) with an anti-acetyl-α-tubulin antibody. A previous report also shows that depletion of *atat* completely removes acetylated α-tubulin signaling in the peripheral sensory neurons [23]. Together, we conclude that Atat is the major α-tubulin K40 acetyltransferase in the nervous system.

### The microtubule networks of muscle cells are largely normal in *atat* mutants

Because their large cell size allows clear visualization of the microtubule network, the *Drosophila* larval muscle system has been used to characterize the functions of microtubule-regulating proteins ☯26, 27, 31]. Disruption of the microtubule chaperon E (TBCE) dramatically decreases microtubule density ☯26], whereas depletion of microtubule severing proteins Katanin 60 [31] increases microtubule density. Previous studies have shown that the levels of K40 acetylated α-tubulin can be reduced by either the overexpression of *HDAC6* [14] or *α1-tubuli* ^*K40Q*^ mutation [27], however, this does not affect the density of the microtubule network, suggesting that acetylation of α-tubulin is not required in muscle cells for microtubule network density. The *atat*^*15*^ and *atat*^*16*^ mutations, which remove the acetylated α-tubulin signal in the muscle cells completely (**Fig 2A and 2B**), did not alter microtubule density (**Fig 2C**). Together, these results support that the acetylation modification of α-tubulin is not required for maintaining the density and organization of microtubule networks in muscle cells.

### Atat is not the sole α-acetyltransferase that regulates microtubule K40 acetylation in adult testes

Mutation in mouse *atat* completely removes the α-tubulin acetylation signal from multiple tissues, including testes [19], suggesting that Atat is the sole α-acetyltransferase in mice. Interestingly, in *atat* mutants acetylated α-tubulin signals in the ovaries of female flies were undetectable, while some acetylated α-tubulin signals remained in the testes of male flies. This indicates that other α-tubulin acetyltransferases may contribute to α-tubulin acetylation in the testes besides Atat (**Fig 3**). Other α-tubulin acetylase homologs, *CG3982* or *CG17003*, in the fly genome have been reported to be expressed in the testes [38, 39], and may mediate the α-tubulin K40 acetylation seen in *atat* mutant testes.

We previously generated two *α1-tubulin* (*α-tubulin* at 84B, the major *Drosophila α-tubulin*) mutations, in which *α1-tubulin* K40 was substituted with glutamine (K40Q) or arginine (K40R) to mimic acetylated or non-acetylated tubulin, respectively [27]. The *α1-tubulin*^*K40Q*^ and *α1-tubulin*^*K40R*^ mutations completely abolished the acetylated α-tubulin signal in the nervous system and muscle cells [27]. However, these two mutations only partially reduced the acetylated α-tubulin signals in the ovaries and testes (**Fig 3**). These results suggest that α1-tubulin is not the only α-tubulin that generates the microtubule network in the ovaries and the testes. The residual signal is probably from the K40 acetylation of another α-tubulin isotype, αTub84D [40].

In summary, our results demonstrate that, unlike in other tissues, multiple α-acetyltransferases are present in adult testes; and the microtubule networks in both ovaries and testes are constructed by multiple α-tubulin isotypes.

### K40 α-tubulin acetylation regulates the morphology of class I dorsal dendrite arborization (da) neurons

The growth of individual tissues during animal development is coordinated with whole-body growth. At the level of sensory neurons, dendrite arbors must grow proportionally with their receptive field to achieve proper wiring. The fly da sensory neuron has been used as an *in vivo* model to study molecular mechanisms of dendritic morphogenesis [41]. Several microtubule-related proteins have been found to play important roles in regulating the morphology and functions of dendrites during development [23, 31, 40–43]. We examined whether Atat-mediated tubulin acetylation regulates dendritic development.

The da neurons can be subdivided into four distinct morphological classes according to their dendritic branching complexity and pattern, ranging from class I neurons with the simplest dendritic arbors to class IV neurons with the most highly branched dendritic trees [33]. Recent studies have shown that *atat* mutations mildly reduce the number of dendrite branch points of the class IV da neurons [23] but blocking α-tubulin acetylation by K40A mutation causes an increased number of dendritic branches [40]. This inconsistency led us to examine the roles of Atat and α-tubulin K40 acetylation in regulating the dendritic morphology of another type of sensory neurons, the class I da neurons, which have much simpler dendritic arbors than class IV da neurons.

In class I da neurons, α-tubulin was acetylated, and the acetylated α-tubulin signals were visually depleted by *atat* mutation (**Fig 4A and 4B**), demonstrating that Atat is the major α-acetyltransferase in class I da neurons. Dendrites of da neurons maintain their coverage to match the growth of the body wall by branching and lengthening during development. We therefore quantitatively analyzed the dendritic field area, dendritic length, and terminal branch numbers of class I da neurons to assess whether α-tubulin acetylation is required for proper dendritic expansion. Dendrites of class I da neurons can be visualized by expressing a membrane-tethered GFP (mCD8-GFP) using *221-Gal4* ☯33]. We found a significant reduction of the dendritic field area covered by the class I da neurons in both *atat*^*15*^ and *atat*^*16*^ mutants, which was fully restored by the re-expression of Atat in class I da neurons (**Fig 4C and 4D**). A slight but not significant decrease in the area of the dendritic field was also observed in mutants carrying the *α1-tubulin*^*K40R*^ mutation (mimicking non-acetylated tubulin) (**Fig 4C and 4D**). The *α1-tubulin*^*K40Q*^ mutation (mimicking acetylated tubulin) had no effect on the morphology of class I da neurons (**Fig 4C and 4D**). We found a slight decrease in total dendritic length only in *atat*^*16*^ mutants (**Fig 4C and 4E**). Neither *atat* mutations nor *α1-tubulin*^*K40*^ mutations affected the terminal numbers of dendrites of class I da neurons (**Fig 4C and 4F**). Together, these results suggest that tubulin de-*acetylation* mainly reduces the area that the dendrite arbor covers.

### Enhancing microtubule stability restores dendritic defects caused by *atat* mutations

**Fig 5:**
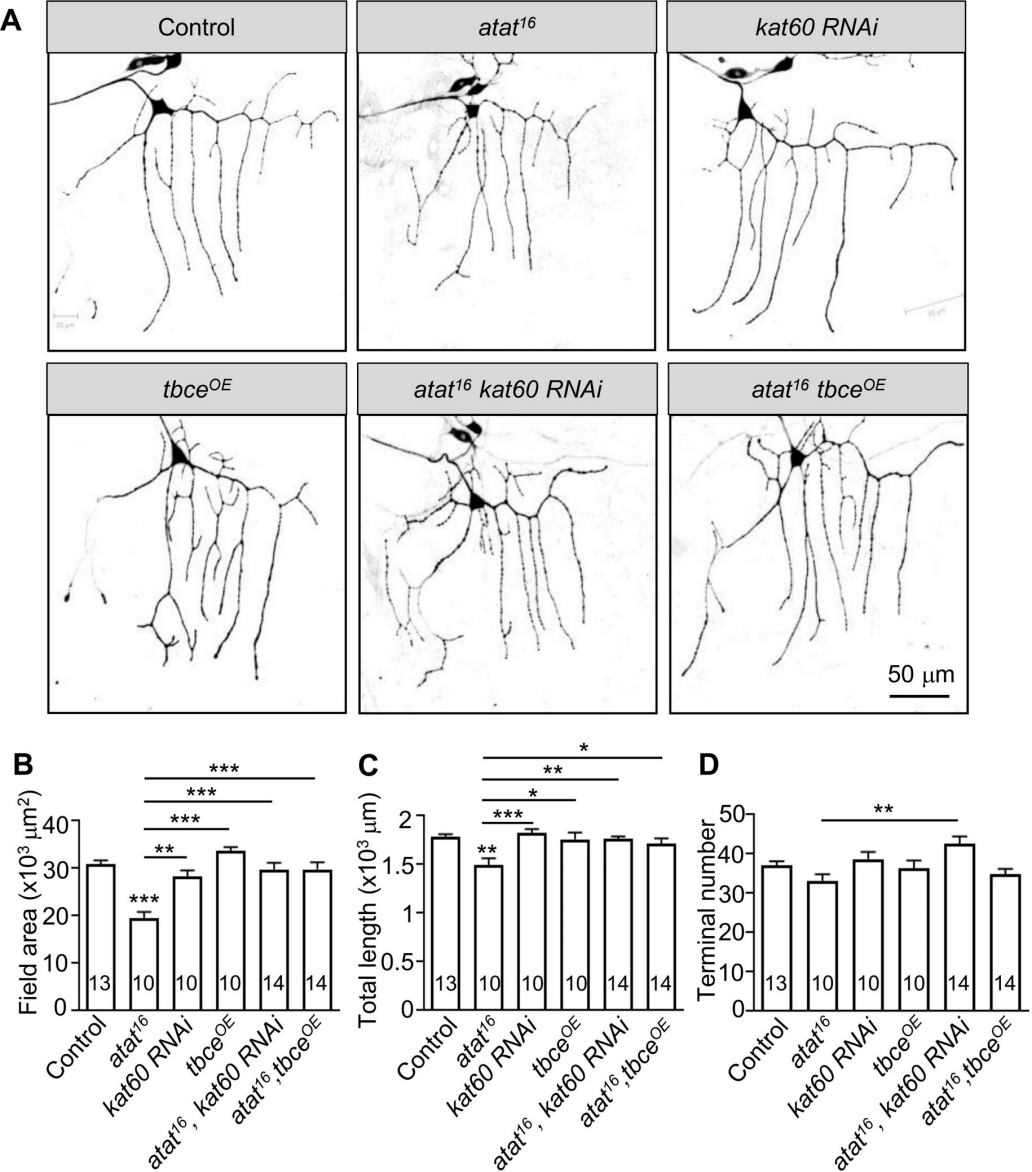
Increasing microtubule stability rescues the dendritic defects caused by *atat* mutation. (A) Dendritic elaboration of class I da neurons labeled with mCD8-GFP under the control of *221-Gal4*. The genotypes are the control (*221-Gal4*>mCD8-GFP), *atat*^*16*^, *katanin 60* knockdown using *221-Gal4* (*kat60 RNAi*), *tbce* overexpression using *221-Gal4* (*tbce*^*OE*^), *katanin 60* knockdown in *atat* background (*atat*^*16*^, *kat60 RNAi*), and *tbce* overexpression in *atat* background (*atat*^*16*^,*tbce*^*OE*^). Scale bar: 50 μm. (B–D) Quantification of total dendritic field areas (B), dendritic lengths (C), and the numbers of dendritic termini (D). Mean ± SEM. One-way ANOVA with Tukey’s multiple comparisons test. ***p < 0.001, *p < 0.05.

Stable microtubules are critical for sustaining dendrite extension and arborization [44]. The acetylation of α-tubulin is associated with stable and long-lived microtubules and has been thought to increase microtubule flexibility, allowing microtubules to better resist repeated mechanical stresses [5, 6, 45]. We reasoned that the depletion of α-tubulin K40-acetylation in *atat* mutants might reduce the stability of microtubules and cause the reduced dendritic expansion of class I da neurons. If this is the case, enhancing the stability of the microtubule would reverse the morphological defects caused by *atat* mutations. Our previous work showed that knocking down the microtubule severing protein *katanin 60* [31] or overexpressing *tbce* [26] dramatically increases microtubule stability and microtubule network density. Both *katanin 60* knockdown and *tbce* overexpression in class I da neurons increased the dendritic field area and total dendritic length in *atat*^*16*^ mutants (**Fig 5**), suggesting that α-tubulin K40-acetylation promotes dendritic elaboration by increasing the stability of microtubules.

### Depletion of K40-acetylation causes an increase in spine-like dendritic membrane protrusions in class I da neurons

**Fig 6:**
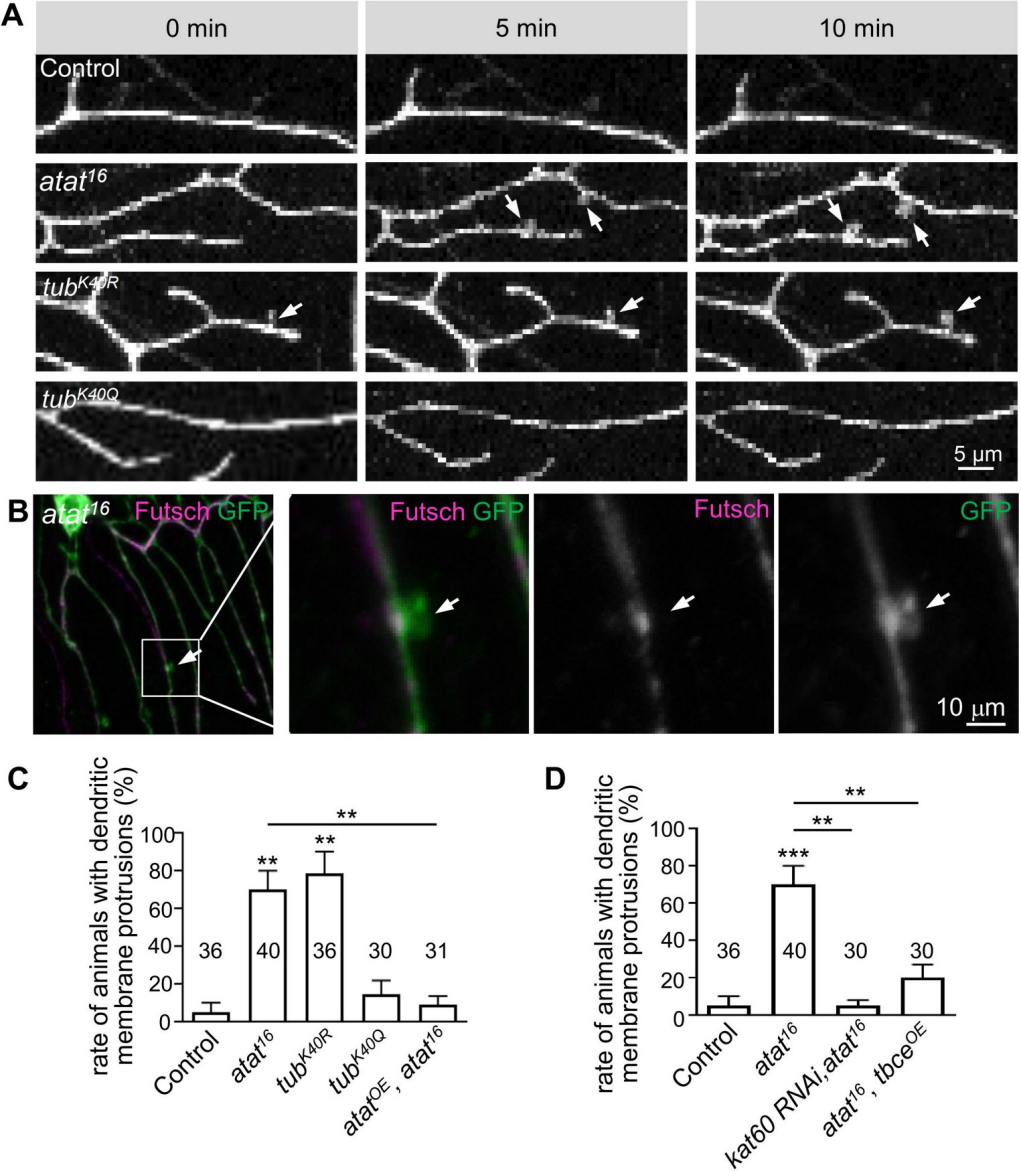
Tubulin deacetylation increases dendritic membrane protrusion formation. (A) Live imaging of dendrites of class I da neurons using the membrane marker mCD8-GFP driven by *221-Gal4* to monitor membrane protrusion formations in second instar larvae of the control (*221-Gal4*> mCD8-GFP), *atat*^*16*^, *α1-tubulin*^*K40R*^ (*tub*^*K40R*^), and *α1-tubulin*^*K40Q*^ (*tub*^*K40Q*^). Arrows denote the protrusions extending from the dendrites. Scale bar: 5 μm. (B) Class I da neurons in second instar larvae of *atat*^*16*^ were stained with anti-Futsch (magenta, labels stable microtubules) and GFP (green). The membrane protrusion (denoted by arrows) lacks Futsch signals. Scale bar: 10 μm. (C and D) Quantification of the percentages of animals with dendritic membrane protrusions. Data were collected from 3~5 independent experiments and 7~15 animals were examined in each experiment. Mean ± SEM. One-way ANOVA with Tukey’s multiple comparisons test. ***p < 0.001, *p < 0.05.

To better understand how Atat regulates the growth of dendrites, we conducted an *in vivo* time-lapse analysis of dendritic structure changes on class I da sensory neurons in live and intact animals. Second instar larvae expressing *mCD8-GFP* using *221-Gal4* were imaged using confocal microscopy for 10 min. The dendritic branches in both control and *atat* mutant larvae at this stage were stable with no obvious branch extension or retractions over the 10-min time interval. Unexpectedly, we frequently found spine-like protrusions in the dendrites of *atat* mutants, which were rarely seen in control animals (**Fig 6A and 6C, S1 and S2 Videos**). Re-expressing Atat in class I da neurons reduced the frequency of dendritic protrusions to the levels of controls (**Fig 6C**). These dendritic protrusions in *atat* mutants often appeared in the secondary and tertiary branches adjacent to the arterial branch of class I da neurons (**Fig 6A, S2 Video**). They were gaining size during the imaging period (**Fig 6A**), were devoid of stable microtubules marked by the microtubule-associated protein Futsch (**Fig 6B**) [46], and morphologically resembled the spine-like protrusions that are abundant in class III da neurons [33]. These dendritic protrusions were likely to be unstable and immature dendritic precursors that were not able to develop into mature branches since we did not see an increase in terminal numbers in *atat* mutants.

Animals carrying the non-acetyl-mimic K40R mutation, but not the acetyl-mimic K40Q mutation, also dramatically increased the chance to have dendritic protrusions in class I da neurons (**Fig 6C**), demonstrating that tubulin K40 acetylation by Atat limits dendritic protrusion formation in class I da neurons. Moreover, stabilizing microtubules by either knocking-down *katanin 60* or overexpressing *tbce* almost fully suppressed the occurrence of dendritic protrusions in class I da neurons (**Fig 6D**). Together, these results suggest that K40-acetylation by Atat may prevent the formation of immature branches in class I da neurons during dendritic development, probably through increasing the stability of microtubules. It would be interesting to investigate the identities and fate of these dendritic protrusions in future studies.

### Tubulin K40 acetylation in class I da neurons regulates larval locomotion

**Fig 7:**
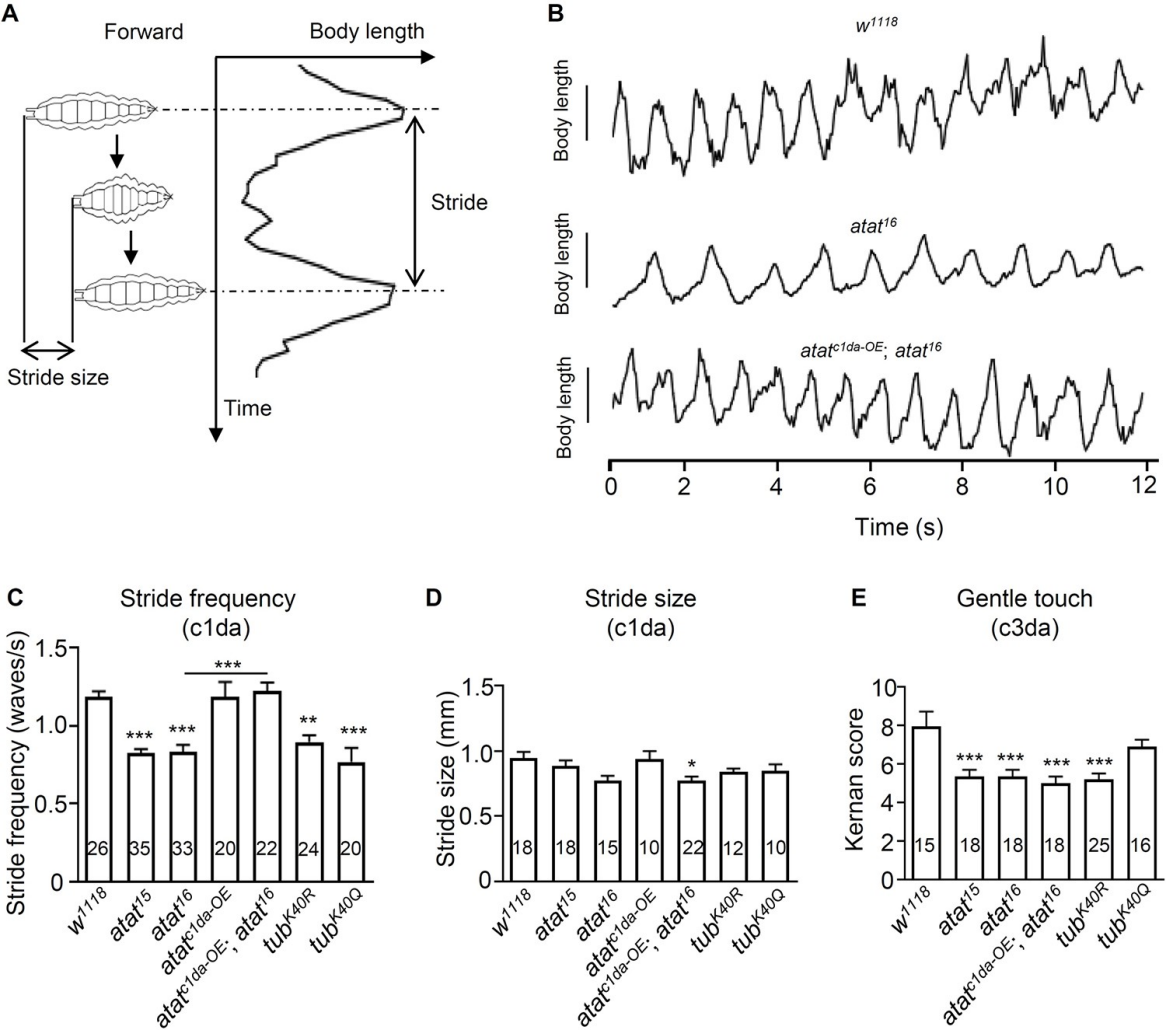
Tubulin K40 acetylation is required for larval locomotor control. (A) Schematic shows the peristaltic contractions during larval crawling. Stride frequency is defined as the number of peristaltic waves of muscle contraction per second, while stride size is the change of body length during one peristaltic wave. (B) Plots of larval body length (mm) over time (s) during larval crawling. *atat*^*c1da-OE*^,*atat*^*16*^: Atat overexpression by *221-Gal4* in *atat*^*16*^ background. (C–E) Quantifications of the stride frequencies (C), the stride size (D), and gentle touch responses (E). Genotypes: control (*w*^*1118*^), *atat*^*15*^, *atat*^*16*^, Atat overexpression using *221-Gal4* (*atat*^*c1da-OE*^), Atat overexpression using *221-Gal4* in *atat*^*16*^ mutant (*atat*^*c1da-OE*^; *atat*^*16*^), *α1-tubulin*^*K40R*^ (*tub*^*K40R*^), and *α1-tubulin*^*K40Q*^ (*tub*^*K40Q*^). Mean ± SEM. One-way ANOVA with Tukey’s multiple comparisons test. ***p < 0.001, *p < 0.05.

We next examined whether Atat-regulated K40 acetylation is required for the proper functions of class I da neurons. During larval movement, class I da neurons provide sensory feedback to the central nervous system that allows coordinated body movements [35, 47, 48]. Genetic silencing of class I da neurons and bipolar dendritic neurons impairs the propagation of waves of muscle contraction [25]. Therefore, we examined the crawling gait of larvae with *atat* or *α1-tubulin*^*K40*^ mutations. Larval crawling consists of repeated cycles of motion called strides (**Fig 7A and 7B)**. We found that *atat* mutations significantly reduced the stride frequency of larval crawling, which were reversed by re-expressing Atat in class I da neurons (**Fig 7B and 7C**), suggesting that Atat is crucial in class I da neurons for setting locomotion kinematics. Mutants carrying the *α1-tubulin*^*K40R*^ mutation also exhibited a decrease in stride frequency (**Fig 7C**), supporting the role of α-tubulin acetylation in regulating larval locomotion. Mutations in *atat* or *tubulin* did not affect the stride size of larval crawling (**Fig 7B and 7D**), which is consistent with previous reports that class I da neurons mainly regulate the frequency but not the size of the strides [35]. We noticed that the reduction of stride frequency in *atat* and *α1-tubulin*^*K40R*^ mutants were smaller than depleting the mechanosensitive TRP channel NompC in class I da neurons [34], indicating that the functions of these neurons are only partially impaired when tubulin acetylation is depleted.

In contrast to class I da sensory neurons that act as proprioceptor neurons, class III da sensory neurons control the behavioral responses to gentle touch stimuli [23, 36]. It has been shown that Atat-mediated tubulin acetylation in class III da neurons increases the sensitivity of touch perception [23]. Consistent with this notion, we found our *atat* and *α1-tubulin*^*K40R*^ mutants were less sensitive to gentle touch (**Fig 7E**). Re-expressing Atat in class I da neurons failed to restore the touch sensitivity defects in *atat* mutants (**Fig 7E**), further supporting that Atat is required for gentle touch sensitivity specifically in class III da neurons. Together, these results suggest that Atat and K40 acetylation are necessary for proper functions of both class I and class III da neurons; depleting *atat* results in impaired functions of both types of da neurons and leads to distinct behavioral defects.

## Discussion

### Tubulin K40 acetylation tunes the development and function of dendrites

Microtubules are essential structural components of dendrites, and various signaling pathways regulate dendritic development by affecting microtubule dynamics [26, 27, 41, 43, 49–51]. Microtubule PTMs, including acetylation, tyrosination, phosphorylation, polyglutamylation, and polyglycylation, are thought to adjust the dynamics and stability of microtubules [52]. PTMs also affect the interactions of microtubules with other cellular components, further contributing to the precise regulation of neuronal microtubule network dynamics ☯52]. Though tubulin acetylation is found preferentially enriched in axons than in dendrites [53, 54], we and others have shown that microtubule acetylation is present in the dendrites of sensory neurons [23, 40] and is important for the development and function of dendrites [23, 40, 55]. An elegant study from the Parrish lab found that acetylated microtubules in dendrites of class III and class IV da sensory neurons are more resistant to mechanical forces, enabling them to convert cellular mechanics to the activation of transient receptor potential channel NOMPC and mechanosensation [23]. Here we show that Atat and α-tubulin K40 acetylation promote the dendritic arborization of class I da sensory neurons (**Fig 4**) and ensure that they process proprioceptive information accurately (**Fig 7**), suggesting that tubulin acetylation is broadly required in the dendrites of multiple types of da sensory neurons for sensory transduction. A similar requirement of tubulin acetylation in dendrites has been found in mammals as well. For example, inhibiting the mouse N-acetyltransferase ARD1-NAT1 complex, which co-localizes with microtubules and promotes tubulin acetylation, impairs dendritic extension in cultured neurons [55]. Thus, tubulin acetylation plays an equally important role in dendrites as it does in axons.

### Tubulin K40 acetylation promotes microtubule stability

It has long been observed that acetylation is enriched in stable and long-lived microtubules that are resistant to depolymerizing drugs [56–58]. However, the stabilization of cellular microtubules also increases acetylation levels [57]. Thus, it was unclear whether acetylation increases microtubule stability or merely labels stable microtubules. Until recently, more evidence supported the direct role of tubulin K40 acetylation in regulating microtubule stability. Two studies from the Nachury lab found that tubulin acetylation reduces lateral interactions between protofilaments thus enhancing microtubule flexibility and protecting long-lived microtubules from mechanical breakage [5, 6]. These findings are further supported by high-resolution cryo-electron microscopy reconstruction, which shows that the acetylation of tubulin restricts the motion of the flexible loop that contains K40 and weakens lateral contacts [59].

Studies in animal models also suggest that tubulin acetylation stabilizes microtubules *in vivo*. Genetic ablation of Atat in nematodes reduces tubulin acetylation and decreases microtubule stability in axons [18]. In *Drosophila*, a lysine-to-alanine mutation of K40 reduces the levels of stable microtubules in the dendrites of sensory neurons [40]. The mutation of *atat* in *Drosophila* also causes the loss of tubulin acetylation and renders the microtubules easier to be broken after mechanical stimuli [23]. In the mouse cerebral cortex, depletion of tubulin acetylation by the ablation of Atat leads to an increase in microtubule unbundling and microtubule plus-end dynamics, which can be reversed by Taxol treatment [20]. Here we show that enhancing the stability of microtubules by the knockdown of *katanin 60* or by overexpressing *tbce* rescues the dendritic defects caused by *atat* mutation (**Fig 4**), providing further support that tubulin acetylation promotes microtubule stability.

Interestingly, decreasing the levels of α-tubulin acetylation by mutating *atat* (**Fig 2)** and *α-tubulin*^*K40*^ or by overexpressing the tubulin deacetylase *HDAC6* does not impair the overall morphology of microtubule networks in larval muscle cells, even though microtubules are heavily acetylated [27]. These observations may indicate that α-tubulin acetylation plays a more important role at the location where microtubule dynamics are highly required and precisely regulated, for example, where newly axonal or dendritic branches are formed.

It should also be noted that tubulins can be acetylated at various sites in addition to K40 [60] and they may have distinct roles in regulating the dynamics of microtubules. Similar to K40 acetylation, α-tubulin acetylation at K394 in *Drosophila* increases microtubule stability and regulates the growth of axonal terminals of motor neurons [42]. In contrast, the tubulin acetylation activity of an N-terminal acetyltransferase, Mnat9, is not required for its function in promoting microtubule stability and cell survival in *Drosophila* [61], indicating that Mnat9-mediated tubulin acetylation has an unidentified role rather than regulating microtubule stability.

### Tubulin K40 acetylation restricts the formation of dendritic protrusions in class I da neurons

Our study reveals that loss of tubulin acetylation either by depleting Atat or by *α1-tubulin*^*K40R*^ mutation does not change the number of dendrites, but dramatically increases dendritic protrusions in class I da neurons (**Fig 6**). These protrusions are probably immature dendritic precursors since they lack stable microtubules and are unable to fully develop into new branches. Enhancing the stability of microtubules by depleting *katanin 60* or the overexpression of *tbce* eliminated most of the extra protrusions in *atat* mutants, suggesting that microtubule destabilization caused by tubulin deacetylation in *atat* and *α1-tubulin*^*K40R*^ mutants increase the number of immature dendritic precursors.

The formation of dendrites requires coordinated interactions between microtubules and the actin cytoskeleton [62]. The emergence of membrane filopodia or spikes enriched in F-actin often precedes the growth of neurites [63], and enhancing F-actin polymerization by expressing the Rho family small GTPase Rac1 increases filopodia formation [64], supporting the role of F-actin in dendritic branch initiation. These F-actin-rich protrusions are further stabilized by the invasion of microtubules, resulting in new dendrite branch formation [51, 64]. In *atat* and *α1-tubulin*^*K40R*^ mutants, deacetylated microtubules may be hyperdynamic and coordinate with F-actin to promote protrusion formation. These protrusions eventually are retracted, most probably because they lack stable microtubules. This idea is supported by a recent study showing the loss of α-tubulin acetylation by depleting Atat, which decreases the stability of microtubules, leads to an increase in microtubule invasion into filopodia and growth cones, and causes axon over-branching in mammalian axons [20].

### Additional α-tubulin acetyltransferases in adult testes

In humans, a reduction in acetylated α-tubulin acetylation is associated with poor sperm motility [65]. Depleting tubulin acetylation by knocking out *atat1*, the sole α-tubulin acetyltransferase in mice also impairs sperm motility and male mouse fertility [66]. We found that tubulins in the *Drosophila* adult testes are highly acetylated, indicating a conserved role of tubulin acetylation in the male productive system (**Fig 2C**). Intriguingly, some acetylated tubulin signals remain in *atat* mutant testes, suggesting that other acetyltransferases exist in the testes (**Fig 2C**). Since *atat* mutants are fertile (data not shown), the remaining acetylated tubulin catalyzed by unknown acetyltransferases may support the normal functions of the testes. Two uncharacterized genes, *CG17003* and *CG3982*, probably encode the tubulin acetyltransferases that contribute to the remaining acetylated tubulin in *atat* mutants because these two putative tubulin acetyltransferases are conserved with human Atat1. A large-scale RNA sequencing study also found they are highly and specifically expressed in the adult testes [67]. More studies are needed to dissect the functions of these two acetyltransferases in the testes.

## Conclusion

We found that loss of α-tubulin lysine 40 acetylation either by depleting the α-tubulin acetyltransferase Atat or by mutating lysine 40 to a non-acetylatable residue in *Drosophila* decreases the dendritic field area of class I da sensory neurons, increases immature dendritic membrane protrusions and impairs larval locomotion. These defects are probably caused by reduced microtubule stabilization due to α-tubulin deacetylation.

## Supporting information

S1 Video Dendritic membrane protrusion in control. Movie of the dendritic membrane protrusion phenotype in class I da neuron in third instar larva of control.
(MP4)

S2 Video Dendritic membrane protrusion in *atat*^*16*^. Movie of the dendritic membrane protrusion phenotype in class I da neuron in third instar larval of *atat*^*16*^.
(MP4)

S1 Raw images (PDF)
