# Supplementary material for: α-Tubulin acetylation at lysine 40 regulates dendritic arborization and larval locomotion by promoting microtubule stability in Drosophila
Source: PLoS One. 2023 Feb 24;18(2):e0280573. doi: 10.1371/journal.pone.0280573 (PMC9955671; doi:10.1371/journal.pone.0280573)
Supplement: S1 Raw images — (PDF) [file pone.0280573.s003.pdf]

supporting figures

Figure S1

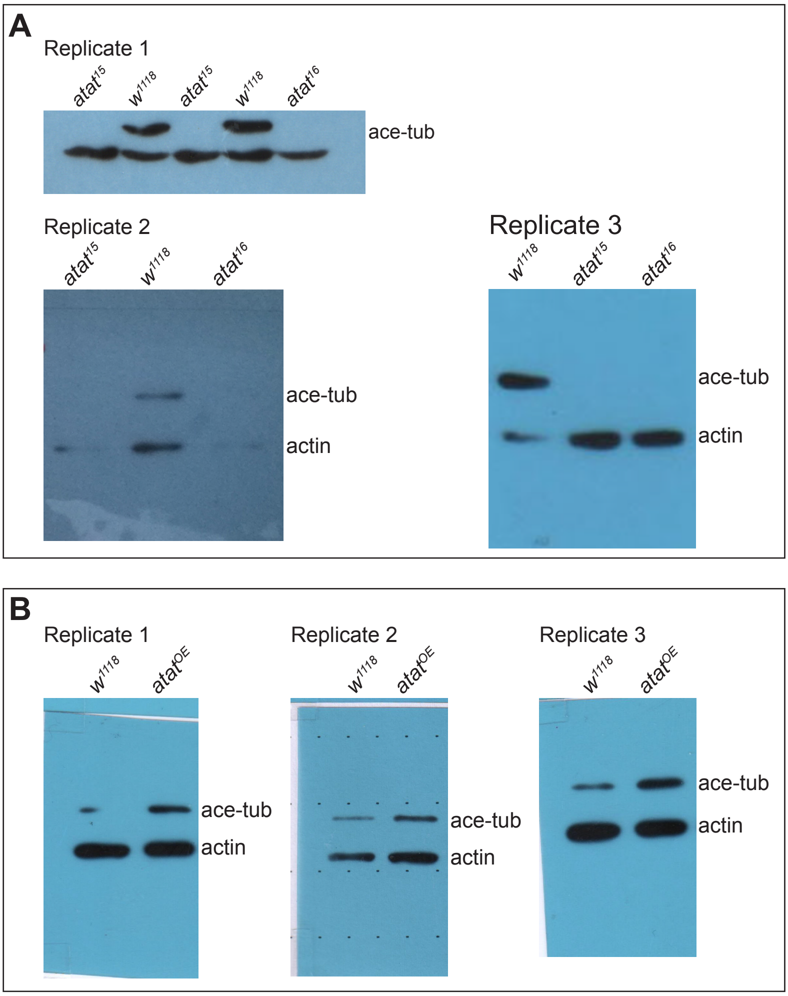

Supporting Figure S1: Western analysis of acetylated α-tubulin in the larval nervous system.

Western analysis of acetylated α-tubulin in the larval nervous system in control (w^{1118}), *atat* mutant (A), and *atat* overexpressed using *elav-Gal4* ($atat^{OE}$) (B). Actin was used as a loading control.
